# Three-year follow-up of the grip concept: an open, prospective, observational registry study on biomechanically calculated abdominal wall repair for complex incisional hernias

**DOI:** 10.1007/s10029-024-03064-2

**Published:** 2024-05-18

**Authors:** R. Nessel, T. Löffler, J. Rinn, F. Kallinowski

**Affiliations:** 1 General, Visceral and Pediatric Surgery, Klinikum Am Gesundbrunnen, Am Gesundbrunnen 20‑26, 74078 Heilbronn, Germany; 2 General and Visceral Surgery, GRN Hospital Eberbach, Scheuerbergstrasse 3, 69412 Eberbach, Germany; 3 General and Visceral Surgery, KKB Hospital Bergstrasse, Viernheimer Strasse 2, 64646 Heppenheim, Germany; 4 Hernia Center, General, Visceral and Transplantation Surgery, University Hospital Heidelberg, Im Neuenheimer Feld 420, 69120 Heidelberg, Germany

**Keywords:** Complex incisional hernia, Biomechanically stable incisional hernia repair, Abdominal wall reconstruction, CRIP, GRIP, Computerized tomography with Valsalva maneuver

## Abstract

**Purpose:**

We studied the effectiveness of biomechanically calculated abdominal wall reconstructions for incisional hernias of varying complexity in an open, prospective observational registry trial.

**Methods:**

From July 1st, 2017 to December 31st, 2020, four hospitals affiliated with the University of Heidelberg recruited 198 patients with complex incisional hernias. Hernias were repaired using biomechanically calculated reconstructions and materials classified on their gripping force towards cyclic load. This approach determines the required strength preoperatively based on the hernia size, using the Critical Resistance to Impacts related to Pressure. The surgeon is supported in reliably determining the Gained Resistance, which is based on the mesh-defect-area-ratio, as well as other mesh and suture factors, and the tissue stability. Tissue stability is defined as a maximum distension of 1.5 cm upon a Valsalva maneuver. In complex cases, a CT scan of the abdomen can be used to assess unstable tissue areas both at rest and during Valsalva’s maneuver.

**Results:**

Larger and stronger gripping meshes were required for more complex cases to achieve a durable repair, especially for larger hernia sizes. To achieve durable repairs, the number of fixation points increased while the mesh-defect area ratio decreased. Performing these repairs required more operating room time. The complication rate remained low. Less than 1% of recurrences and low pain levels were observed after 3 years.

**Conclusions:**

Biomechanical stability, defined as the resistance to cyclic load, is crucial in preventing postoperative complications, including recurrences and chronic pain.

## Introduction

Many people worldwide require surgical treatment for an abdominal incisional hernia every year [1]. Recurrence and chronic pain are significant risks. Around 25% recur within five years. Ten percent of chronic pain is observed [2]. Patients with complex incisional hernias experience more recurrences and higher pain levels. Biomechanically calculated reconstruction (BCR) offers superior outcomes [3].

BCR determines the required strength (critical resistance to impacts related to pressure—CRIP). The surgeon calculates the strength of the designed repair preoperatively (gained resistance—GRIP). The GRIP considers the mesh-defect-area-ratio (MDAR), mesh, suture and other factors [3–5].

We investigated the effectiveness of BCR for incisional hernias of varying complexity. Our analysis is based on a cohort of patients observed prospectively.

## Materials and methods

### Patients

From July 1st, 2017 to December 31st, 2020, four hospitals affiliated with the University of Heidelberg recruited 198 patients for an open observational prospective registry study on complex incisional hernia repair. These patients were included in the Stronghold chapter of the Herniamed® Registry [3].

The Stronghold study is an extension of the Herniamed® registry. Stronghold started in 2017. The aim of the registry is to improve the quality of patient care by monitoring procedures and analysing outcome data. All interested surgeons can easily enter data according to a scientifically validated standard procedure. Patient consent is required [6]. STRONGHOLD follows the same principles as any Herniamed® subset. But it collects seven additional items for biomechanically calculated reconstruction: form of mesh implanted, minimal overlap, number and kind of fixation, pull-out or adapting sutures, type of peritoneal closure and MDAR.

We excluded seven deceased patients from the analysis. The only recurrence is presented and discussed separately. The remainder of 190 patients were classified for complexity. The complexity of incisional hernia repair was evaluated using the Herniamed® approach published in 2021 [2]. The complexity of incisional hernia increases with a defect width above 10 cm, a lateral defect site, a recurrent hernia, age over 80, BMI over 30, comorbidities with adverse metabolic consequences (such as diabetes mellitus), elevated intraabdominal pressure (such as chronic obstructive airways disease), increased risk of bleeding (such as genetic or iatrogenic clot reduction), reduced wound healing (such as concomitant chemotherapy), abnormal gait (for example after a stroke or an amputation), concomitant stoma or intra-abdominal bowel repair (e.g. for the relief of obstruction) and intensified surgery such as component separation. We developed the incisional hernia complexity score by awarding one point for each category. If multiple comorbidity-related risk factors were present, they were combined into a single point.

### Surgical procedures

**Fig. 1:**
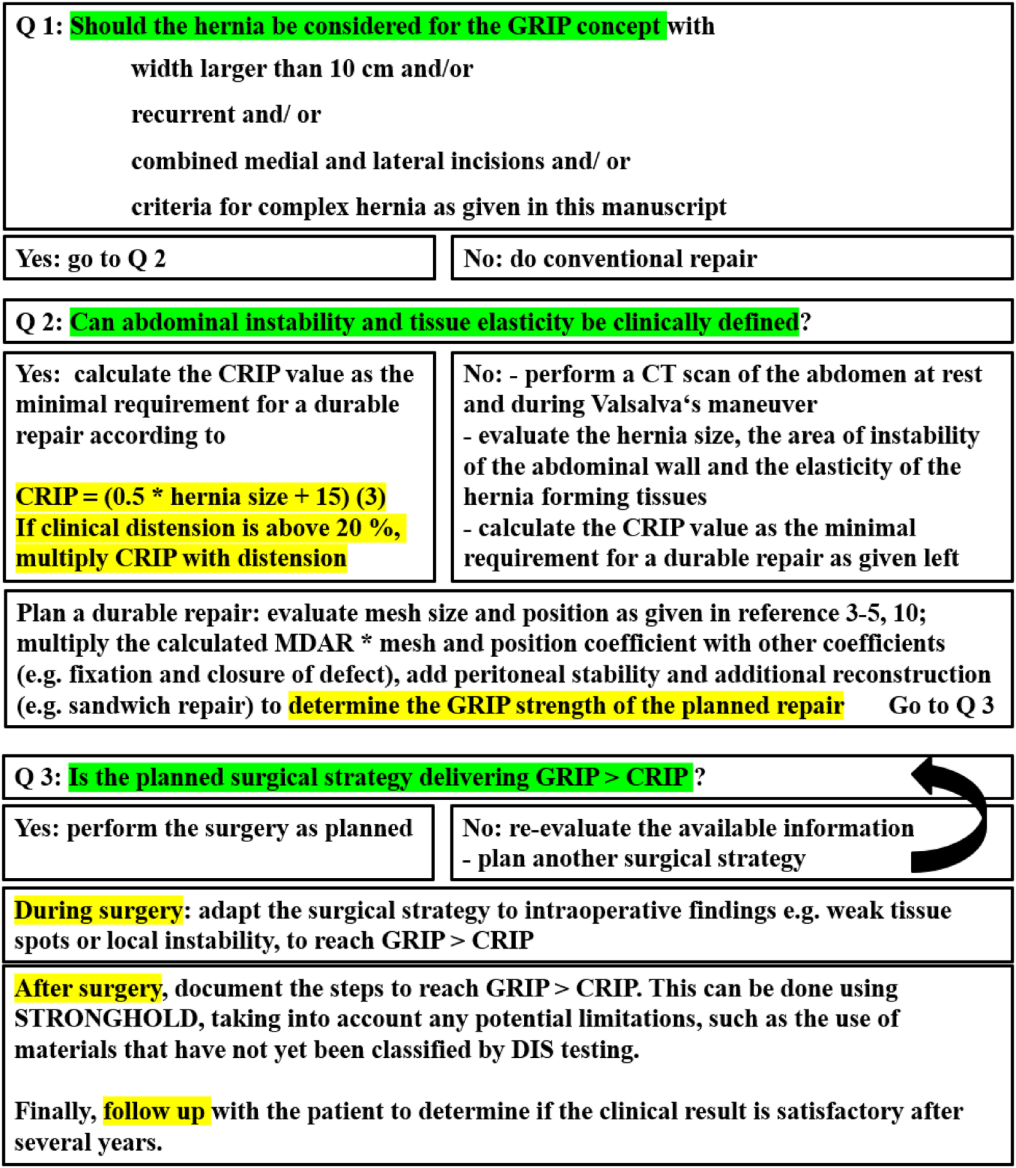
The clinical pathway for biomechanically calculated repair (BCR) involves clinical assessment, abdominal CT with Valsalva if necessary, and calculation of MDAR, CRIP and GRIP values as previously described in references [3–5] and [10]. The process is guided by three questions (Q 1–3). The *arrow* shows the iteration process to reach GRIP > CRIP. The following calculations are included: CRIP = (0.5 * hernia size + 15) * tissue distension [3]. GRIP = MDAR * coefficients for mesh adhesiveness, mesh position in the abdominal wall, number and type of fixation plus factors for peritoneal and fascial closure [3]. For a durable reconstruction, GRIP should be above CRIP

Our hernia repair is based on the concept of biomechanically calculated reconstruction (BCR). The concept is guided by three questions to be answered during the evaluation process (as illustrated in Fig. 1).

BCR yields CRIP and GRIP to guide the design of the surgical procedure. BCR preoperatively determines the required strength, depending on the hernia size, using Critical Resistance to Impacts related to Pressure (CRIP). Mesh-defect-area-ratio, CRIP and GRIP values were calculated as previously described (Fig. 1) [3–5]. GRIP is based on the mesh-defect-area-ratio, mesh and suture factors. The distension of the hernia size and/or the unstable area of the abdominal wall as a measure of tissue stability influences CRIP. Calculating CRIP and GRIP involves four divisions, six multiplications, and one to two additions. It takes approximately five minutes with a pocket calculator. We used a conventional Excel® sheet to determine the hernia and mesh sizes, the number and type of fixation, and the position within the abdominal wall.

**Fig. 2:**
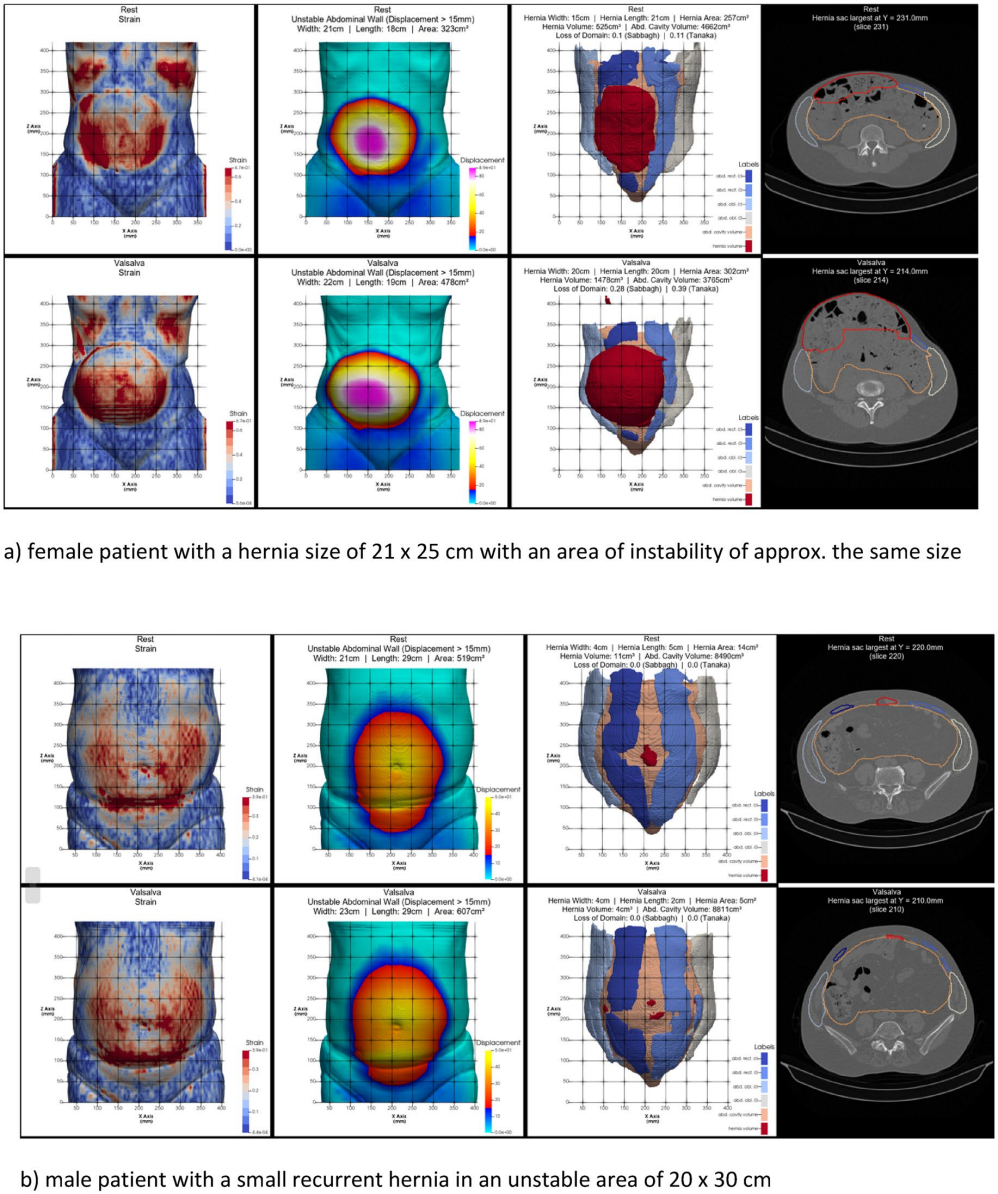

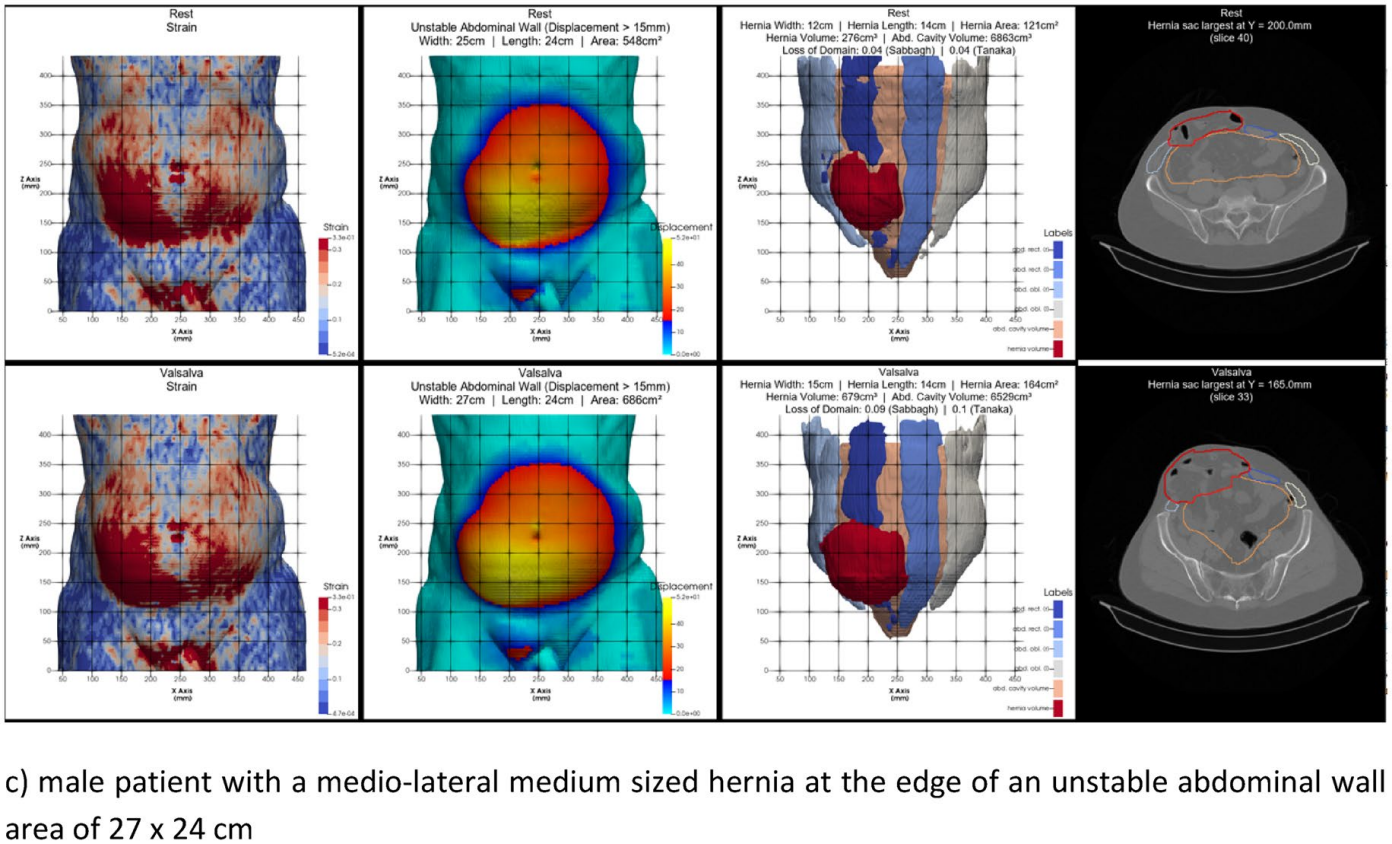
Output of HEDI [8] for 3 patients with a CRIP of approximately 230 during a Valsalva maneuver. For **a**–**c** *top*: at rest, *bottom*: during Valsalva maneuver, from *left* to *right*: strain distribution with higher strain values in red, area of instability > 15 mm starting at the edge of *red* to *blue*, hernia opening (shaded in *dark red*) with musculature (*blue* and *grey*) and mesh landing zone (ochre). The iteration process in Fig. 1 has to take into account the needs to dissipate the energy input by cyclic load and to counteract the anisotropic distension of mesh and tissue. The larger the sizes of the hernia and the unstable wall area, the larger the mesh. More distension increases the need for fixation

Tissue stability was defined as a maximum distension of 1.5 cm during the Valsalva maneuver. In complex cases, unstable tissue areas can be assessed with a CT scan of the abdomen at rest and during Valsalva’s maneuver. To evaluate by hand, three observers must take at least four independent readings of the hernia's width, length, and height. This ensures an interobserver variation of less than 5% [7]. To speed up the process, we developed HEDI [8] as an AI tool to assess tissue stability. HEDI’s evaluation of dynamic computed tomography at rest and during the Valsalva maneuver automatically detects and assesses hernia size, volume and abdominal wall instability. The tool has been in development since the detection of the only recurrence in 2020. Each unstable abdominal wall exhibits a unique strain pattern upon cyclic load (Fig. 2 as an illustration).

DIS class A meshes (Dynamesh® Cicat, Progrip®) with high gripping coefficients were used for complex abdominal wall reconstruction [9]. Most procedures were performed with an open access but MILOS or laparoscopic approaches were also used [4, 5].

The number of intraoperative complications may include bowel lacerations that do not open the internal lining and may be closed with simple sutures. It may also include bleeding requiring hemostatic sutures and unwanted events of any kind. Postoperative complications may include wound or mesh infection or seroma formation, deep vein thrombosis, pulmonary embolism, pneumonia, bleeding, urinary infection, transient or prolonged myocardial or brain ischemia and stroke. Any re-operation within 30 days was recorded.

**Fig. 3:**
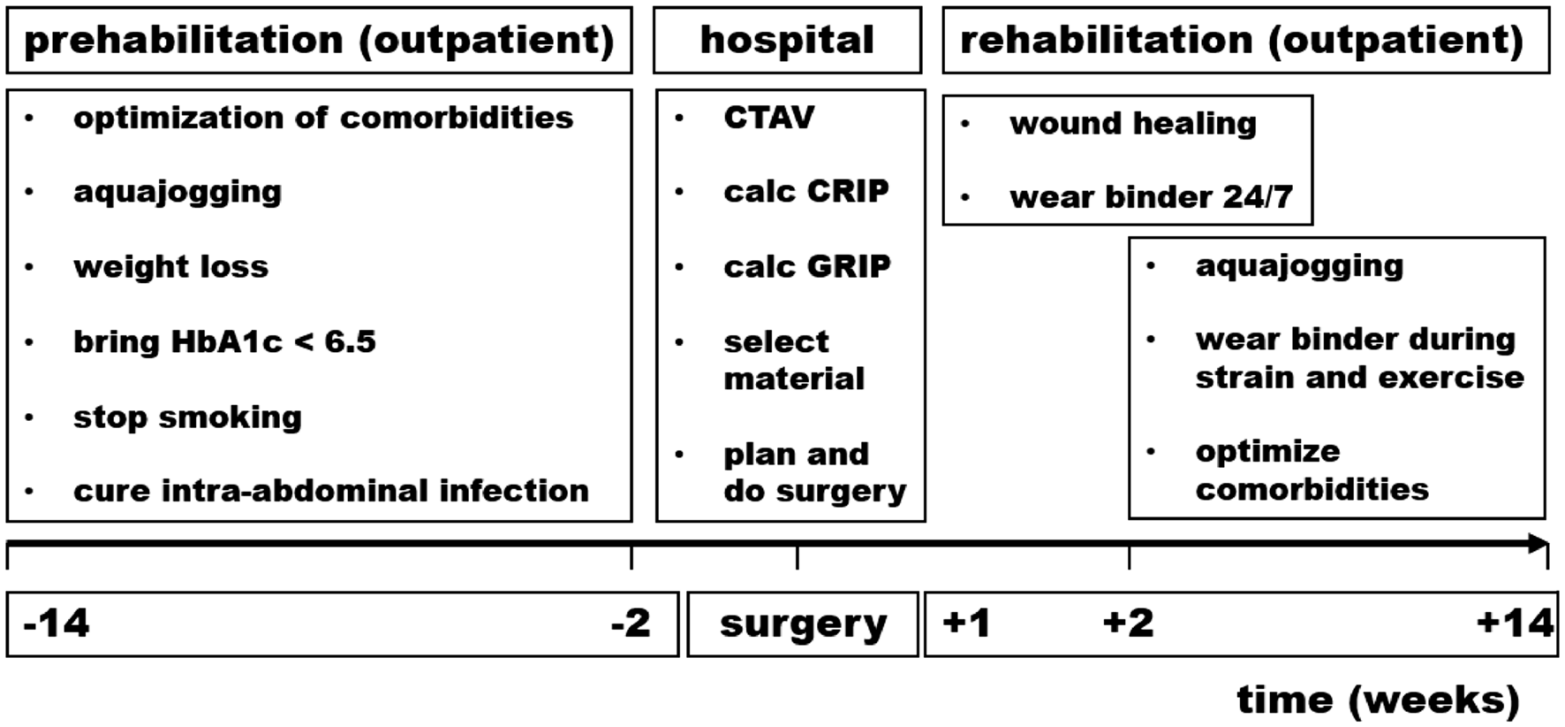
Pre- and rehabilitation before and after BCR. *CTAV* computed tomography at rest and during a Valsalva maneuver, *CRIP* critical resistance to impacts related to pressure, *GRIP* gained resistance to impacts related to pressure. Wounds typically heal within two weeks. To promote stable scar formation, the authors recommend to wear an abdominal binder during wound healing, both day and night, and during physical activity afterwards

Hernia repair was embedded into a pre- and rehabilitation program (Fig. 3).

### Follow up procedures

Patients were regularly followed-up via telephone interviews with themselves, known relatives, or family physicians. During these interviews, patients were asked about any unwanted effects, such as pain at rest or during exercise, that required medication. All re-operations, including imaging and a review of the OR report, were assessed. Patients with bulges on the body were asked to come to the hospital for clinical examination and, if necessary, ultrasonography, magnetic resonance imaging or computed tomography. No patient was lost to follow-up.

### Statistics

Key descriptive statistics were calculated as given in Tables 1–5. As the data were skewed, non-parametric tests (group homogeneity with Kruskal–Wallis, then u-tests if necessary) were evaluated.

**Table 1:**
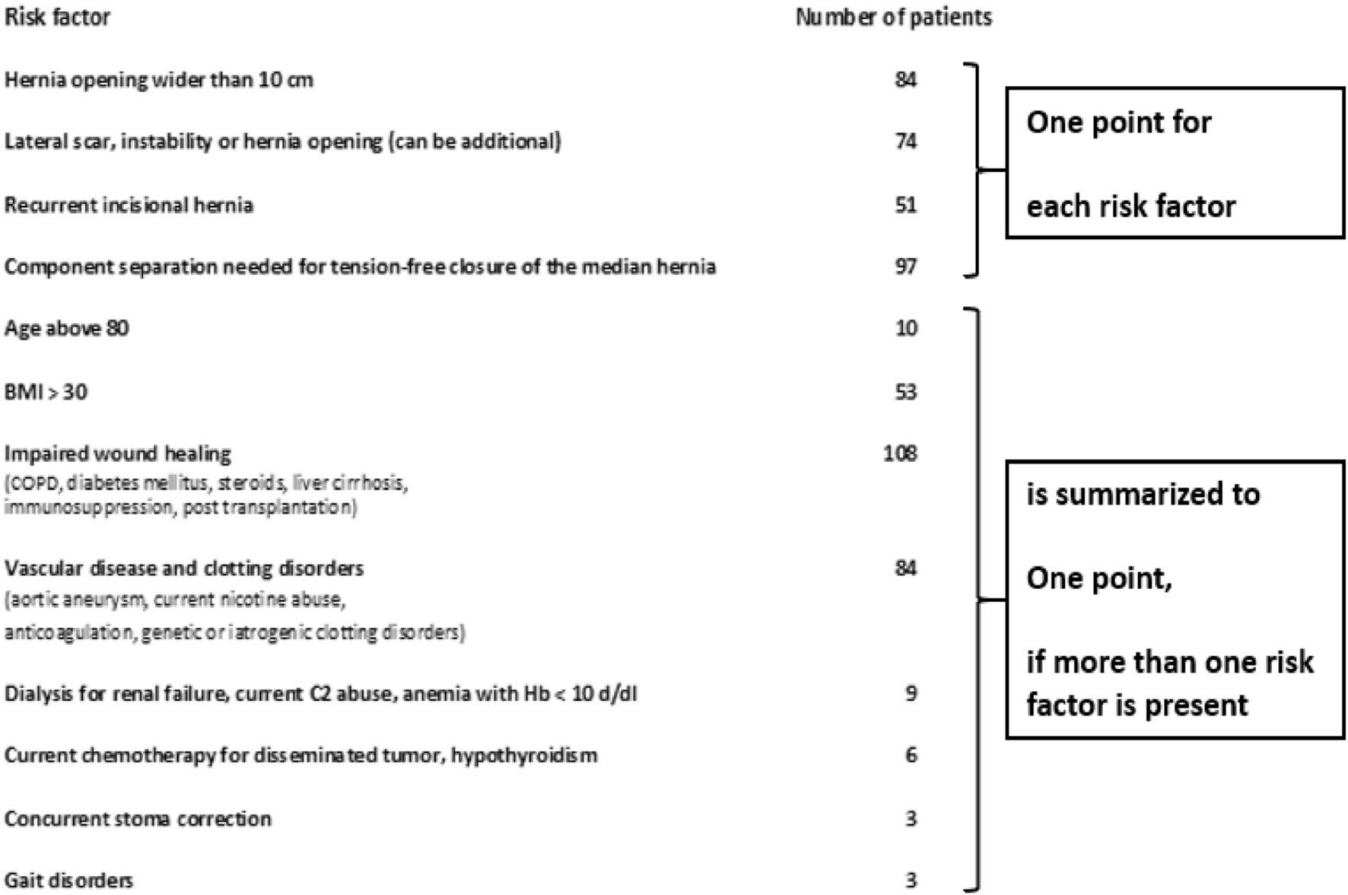
List of risk factors for complex incisional hernia repair

## Results

In the study group without recurrences, 95 women and 95 men had a median age of 64 years (mean ± SD: 63 ± 12, range: 27–92 years). The only recurrence occurred in May 2020, eight months after the initial repair, in a female recipient of liver transplant.

After classification to the new Herniamed® approach, 18 patients no longer underwent a complex incisional hernia repair. Each remaining patient had between one and five risk factors (refer to Table 1).

In the remaining 172 patients, the complexity of the incisional hernia ranged from one to five, as shown in Table 2. Otherwise, the cohorts are comparable.

**Table 2:** Basic condition of patients

Complexity Score	0	1	2	3	4	5
Number of patients	18	44	45	31	34	18
Women	9	26	26	16	10	8
Men	9	18	19	15	24	10
Age (mean ± SD)	61 ± 14	65 ± 13	66 ± 12	63 ± 14	62 ± 14	57 ± 10
Median	64	66.5	67	64	64	55
Minimum	27	32	43	28	33	39
Maximum	79	89	92	81	82	76
BMI (mean ± SD)	25.7 ± 2.5	29 ± 4.8	30 ± 6	27 ± 5	30 ± 6.5	29 ± 5
Median	25.5	29.2	27.8	26.4	27.8	27.8
Minimum	22	19.7	19.8	16.8	20.5	20.7
Maximum	29.3	29.2	45.6	34.3	54.7	38.9
Preop pain (NAS score, mean + SD)	2.4 ± 2.7	2.6 ± 2.1	3.3 ± 2.2	3.8 ± 2.8	4.2 ± 2.6	5.5 ± 3
Median	2	3	3	3	4	6
Minimum	0	0	0	0	0	0
Maximum	10	7	9	8	9	10

Uncomplicated cases with a complexity score of 0 consisted of primary incisional hernias treated electively. As complexity increases, the number of male patients increases, while age and BMI remain constant. The increase in preoperative pain levels tended to coincide with higher complexity. No significant trends were found.

Hernia sizes increased significantly as the complexity increased (*p* < 0.00001). Larger meshes are required to achieve durable repair for larger hernia sizes (*p* < 0.00001; see Table 3). The number of fixation points increased while the mesh-defect area ratio decreased (*p* < 0.00001) to achieve a GRIP value above CRIP. Additional OR time is required to perform durable repair for larger or more complex herniae (*p* < 0.00001).

**Table 3:** Biomechanical parameters

Complexity score	0	1	2	3	4	5
Hernia width (cm, mean ± SD)	4 ± 1	5 ± 2	7 ± 4	11 ± 4	15 ± 5	17 ± 4
Median	4	5	6	10	15	17
Minimum	2	1	2	3	4	11
Maximum	8	11	21	20	30	25
Hernia length (cm, mean ± SD)	6 ± 3	8 ± 6	11 ± 7	15 ± 7	19 ± 7	19 ± 9
Median	5	7	9	17	20	19
Minimum	2	2	2	3	5	7
Maximum	10	26	30	30	39	40
Mesh width (cm, mean ± SD)	15 ± 5	16 ± 4	21 ± 7	27 ± 6	31 ± 6	32 ± 7
Median	15	15	20	30	30	30
Minimum	8	9	8	13	15	24
Maximum	30	30	33	40	45	49
Mesh length (cm, mean ± SD)	20 ± 6	22 ± 7	28 ± 12	36 ± 10	42 ± 6	40 ± 11
Median	20	20	25	39	45	45
Minimum	9	9	12	15	24	19
Maximum	30	45	45	45	45	49
Minimal overlap (cm, mean + SD)	5 ± 1	5 ± 2	6 ± 3	6 ± 2	7 ± 3	5 ± 2
Median	5	9	5	6	6	5
Minimum	3	4	3	2	2	2
Maximum	6	5	15	11	13	10
Mesh-defect area ratio (mean ± SD)	19 ± 12	22 ± 31	14 ± 10	8 ± 5	7 ± 8	5 ± 3
Median	16	11	9	7	5	5
Minimum	4	3	4	2	2	2
Maximum	53	143	40	24	38	14
Number of fixation points (mean ± SD)	25 ± 22	29 ± 31	56 ± 42	79 ± 54	126 + 67	136 ± 70
Median	18	15	50	70	120	130
Minimum	2	4	0	0	20	36
Maximum	70	130	136	257	300	280
CRIP (mean ± SD)	25 ± 7	35 ± 21	48 ± 29	79 ± 35	136 ± 66	147 ± 81
Median	22	28	34	86	122	128
Minimum	17	16	17	20	28	54
Maximum	43	101	139	172	316	329
GRIP (mean ± SD)	254 ± 212	279 ± 498	327 ± 310	273 ± 187	379 ± 373	353 ± 241
Median	165	86	248	239	323	299
Minimum	14	14	15	22	36	57
Maximum	700	2657	1565	781	2296	1071
Length of surgery (min, mean ± SD)	112 ± 36	118 ± 47	159 ± 68	201 ± 61	247 ± 76	290 ± 75
Median	114	106	150	205	240	278
Minimum	47	48	52	103	150	140
Maximum	169	287	360	360	480	420

HEDI was not necessary for less complex repairs. The HEDI output is related to abdominal wall instability. The distorsion field is calculated using a symmetric diffeomorphic registration method [8]. It was first applied in 2% of cases with a complexity score of 2, 6% in group 3, and 9% in group 4. In the most complex cases, one-third of cases were assessed using HEDI. However, since HEDI became available in 2020, the last year of recruitment for this report, this does not reflect the true need. Today, every complex case is evaluated with HEDI before elective repair. This is done to gain insight into biomechanical parameters [8].

In the highest-complexity group, 94% of patients underwent transversus abdominis release augmented with a DIS class A mesh with non-resorbable suture fixation. Furthermore, half of the patients underwent a single crown tack fixation using absorbable tacks. In addition, 44% had a second mesh in the intraperitoneal underlay repair (IPUM as a sandwich, usually with a biosynthetic Phasix® mesh). To counteract a jump of tissue compliance at fascial or bony edges, transmural fully absorbable pull-out sutures and Arthrex® bone anchors were used in 11% and 9% of cases. Area bonding with fibrin glue was used to dissipate the energy of cyclic loading in 6%. The patient with over 80% domain loss and tissue distension exceeding 10 cm was treated with progressive pneumoperitoneum. No botulinum toxin or Fasciotens® was necessary in any case. The calculated GRIP increased from no complexity to complexity level 4 (p = 0.00203) and remained constant thereafter because the GRIP coefficients of these combined procedures for complexity class 5 cases have not yet been determined. Surgical access was open for retromuscular, TAR, and sandwich repair in 82%, MILOS in 11%, and laparoscopic eTAR in 7% of all cases. No robotic procedures were performed in this study. The increase in operation time reflects the increasing complexity of the surgical requirements.

Patients with increasingly complex abdominal wall repairs required a longer hospital stay (*p* = 0.00031, Table 4). There was a tendency for more intra- and postoperative complications with increasing complexity. The rate of reoperations remained constant. Pain at discharge was comparable in all groups and diminished thereafter (Table 5). At the 3-year follow-up, only one patient occasionally took an analgesic, while 189 patients did not take any. All patients under the age of 62 were able to return to work after 14 weeks of rehabilitation. Some of these patients had been on and off work for up to 20 years prior to BCR.

**Table 4:** Outcome parameters

Complexity Score	0	1	2	3	4	5
Length of stay (days)	4.8 ± 2.1	6.1 ± 2.5	7.2 ± 5.5	7.8 ± 3.1	11.3 ± 18.4	13.1 ± 18.6
Median	4.5	6	6	7	7	7
Minimum	2	2	2	5	4	4
Maximum	10	16	36	18	113	70
Number of intraoperative complications	1	1	3	0	1	2
Rate of intraoperaive complications (%)	6	2	7	0	3	11
Number of postoperative complications	0	1	6	4	8	2
Rate of postoperative complications (%)	0	2	13	13	26	11
Number of re-operations	0	1	1	0	2	1
Rate of re-operations (%)	0	2	2	0	6	6
Pain at discharge (NAS score, mean + SD)	2.6 ± 1.9	1.9 ± 1.2	2.4 ± 1.6	2.6 ± 1.4	2.1 ± 1.3	2.5 ± 2
Median	2	2	2	2	2	2
Minimum	0	0	0	0	0	0
Maximum	8	5	6	6	6	6

**Table 5:** Pain related to incisional hernia complexity during follow-up (median NAS scores)

Complexity score	Pain at discharge	After 1 month		After 6 months		After 1 year		After 3 years	
		Rest	Exercise	Rest	Exercise	Rest	Exercise	Rest	Exercise
0	2	1	1	0	0	0	0	0	0
1	2	1	1	0	0	0	0	0	0
2	2	1	2	0	0	0	0	0	0
3	2	1	2	0	0	0	0	0	0
4	2	0	1	0	0	0	0	0	0
5	2	1	2	0	0	0	0	0	0

## Discussion

In an open prospective observational registry trial, we studied the effectiveness of biomechanically calculated abdominal wall reconstructions for incisional hernias of varying complexity. This report expands previous knowledge on biomechanical stability of herniated abdominal walls on a larger patient base [3, 10]. Our study provides insight into complexity-related biomechanical aspects of incisional hernia repair. Our results are positive.

The human abdominal wall consists of different layers of polymers, including the aponeurosis, fascia, and musculature [11]. BCR repairs a defect with a DIS class A textile [3]. Similar to engineering and materials science, cyclic loading is crucial to test the behavior of structural composite. It refers to the application of repeated or fluctuating stresses, strains or stress intensities at specific locations on structural elements. In complex incisional hernia repair as well as in aerospace, automotive, civil engineering, and orthopedics, cyclic loading can cause degradation over time [12].

To develop BCR, two new technologies were necessary [8, 9]. First, biomechanical testing is required using a home-built cyclic loading bench test. This test is now in its fourth generation and includes temperature control, varying tissue stretch, pressure, impact area and other features. Second, HEDI was developed as an AI based tool to assess tissue quality. The HEDI program can be run on a standard computer from 2021 at a cost of US$ 1,500 and is available for free on GitHub.

**Fig. 4:**
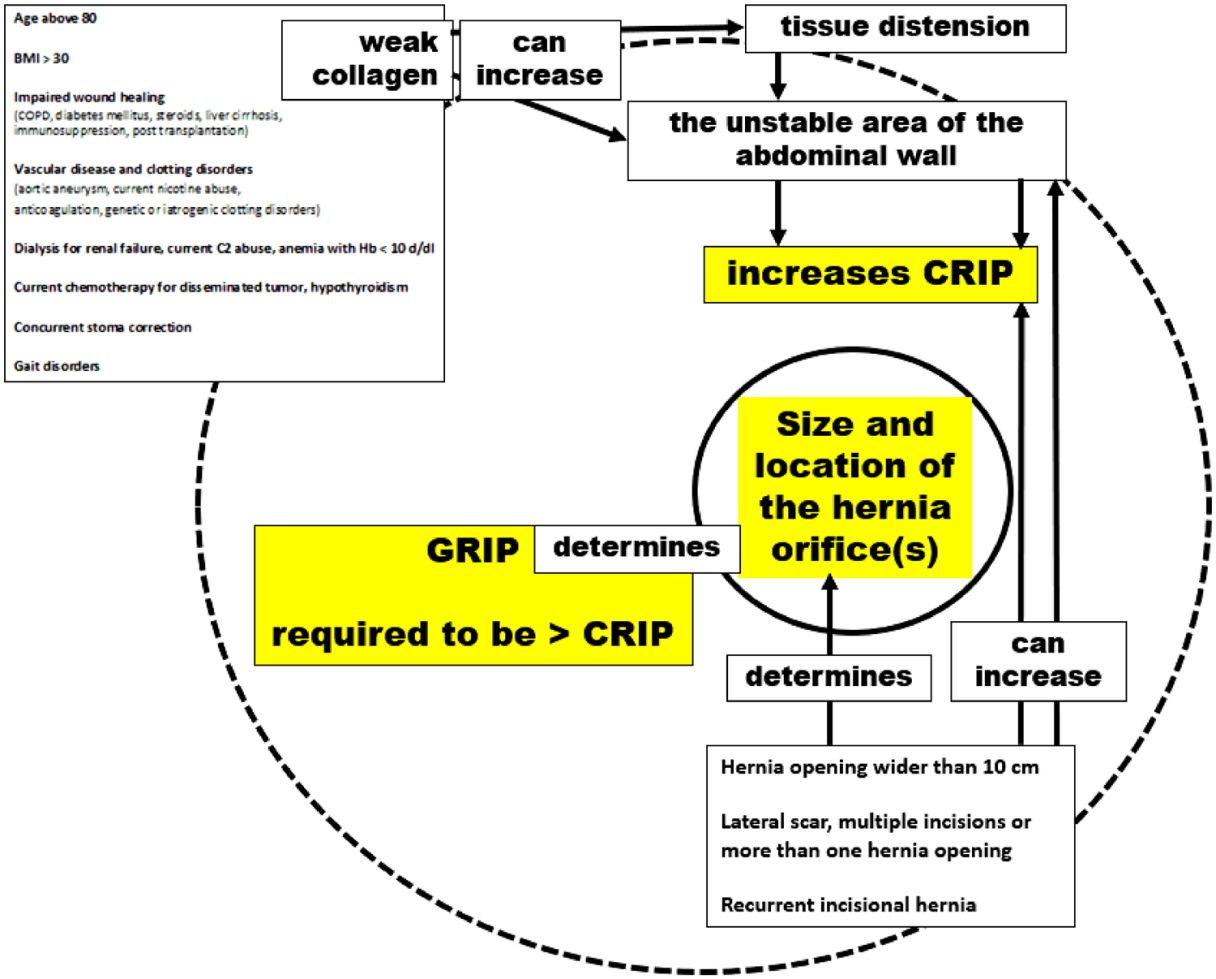
The biomechanical parameters of durable abdominal wall repairs are influenced by complexity and comorbidity. The hernia orifice, located in the center, is often surrounded by an unstable area of the abdominal wall (*solid* and *broken lines*). Weak collagen resulting from comorbidities can increase tissue distension and the unstable area of the abdominal wall, leading to higher CRIP values. Complexity-related factors determine the size and location of the hernia orifice. These factors can cause increased tissue distension and instability (see Fig. 2 for further illustration)

It is important to stabilize unstable wall areas in our biomechanical concept. It is also important to durably close hernia openings. Unstable wall areas, such as rectal diastasis, can occur without a hernia. Hernia openings, such as those in lateral inguinal hernia, can occur without unstable wall areas. In the case of complex incisional hernia, it is necessary to consider both aspects together. The issue of stability revolves around collagen turnover because freshly formed collagen is unstable and requires approximately 84 days for durable cross-linking [13, 14]. Comorbidities affect the time required for collagen formation and stabilisation as well as other tissue components, which can affect the extent of instability. The hernia size is influenced by previous surgical procedures (Fig. 4).

Consideration of the biomechanical principles of cyclic loading enables durable repair of incisional hernias, even in very complex cases [3, 5]. Approximately 50% of our patients have an elasticity of >20% or a shift of >15 mm; therefore, it is important to identify and repair any unstable areas of the abdominal wall [4, 15].

We observed one recurrence in a 64-year-old woman with a 20 cm wide and 32 cm long incisional hernia after liver transplantation under continued immunosuppression. At that time, we calculated the hernia size from four repeated assessments of a CT scan at rest and during a Valsalva maneuver performed by three different observers to achieve less than 5% variation [4, 7]. In this patient, the interobserver variation was 18%. After eight months of follow-up, a recurrence was observed at the right lateral edge of the L-shaped incision. In retrospect, this was caused by a high laxity area, which resulted in a high variation of hernia size assessment. We believe that the recurrence is a consequence of this area.

To address this issue, we developed HEDI, an AI tool that detects unstable abdominal wall [8]. HEDI is now routinely used to identify lax tissue zones with high tissue distortion. We believe that HEDI analysis is superior to computer simulation. Because the effects are directly observed, can be checked by independent observers, and depend only on the power exerted by the patient [16]. Simulation scenarios are often limited by neglecting the effects of cyclic impacts or boundary conditions that do not reflect realistic loads [17].

Our group has published papers on biomechanics to evaluate the underlying shakedown concept in more detail [4, 10, 13]. We found that 15 mm is a good general value for distinguishing between stable and unstable areas; therefore, we use this limit in our clinical work. The latest application of HEDI enables alternative options in millimetre steps, as well as 2D and 3D projection. Further research is required on this topic.

Recently, there have been attempts to classify the complexity of incisional hernia repair. This is done to prioritise patients on waiting lists, assess quality of life, outcomes, recovery, and recurrence rates. New meshes and modern surgical techniques are also considered [2, 18, 19]. Our study is the first to relate biomechanics to the complexity of incisional hernia repair. Our results are positive. We summed all comorbidities into one point owing to their biomechanical effect, which increases tissue laxity (Fig. 2 refer to Fig 4).

Bleeding disorders were thought to have a direct impact on complication rate, but this study shows otherwise. Our CEDAR risk analysis revealed complication risks ranging from 25 to 99%, but complication rates in our study ranged from 0 to 26%, including minor events [20]. Stabilized tissue appears to minimize complications such as seroma or hematoma formation.

Biomechanical stability is crucial to prevent postoperative complications and recurrence. Similar to calcified tissue, soft tissue fusion and healing requires stability [21]. Stabilized tissue exudates briefly followed by collagen formation. Crosslinks form in stable tissue within weeks [13]. These data, our group’s previous publications [3–5, 7–9, 12] and the work presented in [15, 22] were used to develop a clinical pathway (Figs. 1–3).

Abdominal wall instability can lead to burst abdomen and incisional hernia. They may develop within days or weeks after surgery [23]. In a pilot study of 800 patients who underwent major surgery at our hospital, we found that 13% developed incisional hernias after 1 year, with 3% being complex cases (20% of all incisional hernias after one year). Therefore, we recommend secondary prophylaxis: all patients at risk should be assessed within months after surgery, and hernias should be repaired early to avoid complex cases.

Recurrences of incisional hernias occur early but may not become apparent until later, with two-thirds of recurrent incisional hernias becoming apparent after three years [24]. Our study shows that complex incisional hernias can be repaired with very low recurrence rates when considering biomechanical and cyclic loading principles. This study found that after three years, BCR can result in 99% durable repairs of complex incisional hernias.

A complex incisional hernia cannot be repaired by simply using a larger mesh or more fixation [12, 25]. For a stable mesh-tissue interface and for pain-free fixation, it is important to consider the gripping force towards cyclic load [3, 9, 15].

Complex surgeries result in longer surgery times and hospital stays. They require better materials, ultimately resulting in more investment. However, the investment is balanced by the benefit of doing the correct repair the first time around. BCR can help achieve this. Additionally, pain, which increases with complexity, decreases after a biomechanically calculated repair.

## Conclusions

Preoperative calculations of biomechanical stability can guide the surgical design of complex hernia repair. Complexity can be scored related to biomechanics. Durable repairs require materials and OR time that are significantly related to increasing complexity of incisional hernia. Complex incisional hernia can be repaired at very low recurrence and chronic pain rates considering biomechanical and cyclic loading principles. Randomized trials are needed to confirm the advances possible with BCR, as this study provides the first promising long-term results.

## Data Availability

The raw data supporting the conclusions of this article will be made available by the authors, without undue reservation considering restrictions by national and European data protection laws. All data supporting the findings of this study are available within the paper.

## Abbreviations

BCR: Biomechanically calculated reconstruction
CRIP: Critical resistance to impacts related to pressure
GRIP: Gained resistance to impacts related to pressure
BMI: Body mass index
NAS: Numerical-analog scale
MDAR: Mesh-to-defect-area-ratio
HEDI: Hernia evaluation, detection and imaging
AI: Artificial intelligence
MILOS: Mini- or less-open sublay operation
OR: Operation room
DIS: Dynamic intermittent strain
IPUM: Intraperitoneal underlay mesh
CT: Computed tomography
CEDAR: Carolina estimate of disease adjusted risk
HbA1c: Glycosylated hemoglobin type A1c
CTAV: CT abdomen at rest and during a Valsalva maneuver
